# Oxymatrine relieves high-fructose/fat-induced obesity via reprogramming the activity of lipid metabolism-related enhancer

**DOI:** 10.3389/fendo.2023.1145575

**Published:** 2023-08-04

**Authors:** Luping Ren, Xuehua Liu, Xitong Huang, He Zhang, Wenjie Fei, Xian Yu, Zhijuan Hu, Yunfeng Zhen, Shuchun Chen

**Affiliations:** ^1^ Department of Endocrinology, Hebei General Hospital, Shijiazhuang, Hebei, China; ^2^ Graduate School of Hebei North University, Zhangjiakou, Hebei, China; ^3^ Department of Traditional Chinese Medicine, China Pharmaceutical University, Nanjing, Jiangsu, China

**Keywords:** obesity, oxymatrine, Smad3, enhancer, fatty liver disease

## Abstract

**Introduction:**

Emerging evidence demonstrates that the high-fructose and high-fat diet (HFHF) induced obesity and fatty liver disease has become one of the most common metabolic disorders worldwide. Therefore, innovative investigations on compounds targeting obesity and fatty liver diseases are urgently needed.

**Methods:**

The high-throughput natural compounds screen was performed to screen the important compounds. A rat HFHF model was constructed, the regulatory function of Oxymatrine in HFHF-induced obesity was further explored.

**Results:**

We identified Oxymatrine, a natural compound extracted from *Sophora flavescens*, showed a potential compacity in high-fat diet-induced fatty liver disease. We found that oxymatrine significantly inhibited HFHF-induced obesity using a rat HFHF model. Additionally, we found that oxymatrine altered the enhancer landscape of subcutaneous adipose tissues by ChIP-seq analysis using antibodies against the H3K27ac histone modification. Motif enrichment analysis showed the Smad motif was significantly enriched in enhancers altered post-oxymatrine treatment. Further chromatin immunoprecipitation-quantitative PCR (ChIP-qPCR) analysis and luciferase reporter assays showed oxymatrine alters the binding of Smad3 on the enhancer regions of B-cell lymphoma 2 (Bcl2) and the enhancer activity of Bcl2.

**Discussion:**

Together, our study highlighted oxymatrine could suppress high-fructose and high-fat diet-induced obesity by inhibiting the suppressor of mothers against decapentaplegic 3 (Smad3) binding on obesity-related enhancers.

## Introduction

Food and beverages that contain high energy, fat, and/or sugar are recognized as one of the significant causes of obesity and metabolic disorder, in addition to genetic predisposition and physical inactivity (1, 2). The typical western diets are now generally characterized by an overabundance of saturated fats and carbohydrates. Carbohydrates in such diets typically contain more monosaccharides (such as glucose and fructose) and disaccharides (such as lactose and sucrose) than complex carbohydrates (3). For instance, fructose is widely used in desserts and beverages but is evidenced in increasing the risk of dyslipidemia, obesity, type 2 diabetes, and heart disease (4). High-fructose and high-fat diet (HFHF)-induced obesity and metabolic disorder are emerging healthy challenges (5).

In conjunction with a healthy diet and physical activity, some medications have achieved success in the treatment of HFHF-induced obesity and associated diseases. Recently, the application of natural products shows a potential therapeutic effect as an alternative treatment strategy for metabolic disorders such as Oxymatrine, Taxifolin (6), Naringin (7), Corylin (8), etc. We and other have also found that Oxymatrine, a natural compound extracted from *Sophora flavescens*, have a potential therapeutic effect in many diseases, including metabolic disorder-related disease (such as fatty liver disease). Oxymatrine inhibited the transcription factor sterol regulatory element binding transcription factor 1 (SREBF1) and Peroxisome Proliferator-activated Receptor α (PPARα) and suppressed hepatic steatosis in non-alcoholic fatty liver disease (9, 10). In addition, Oxymatrine significantly increased Sirt1 expression and AMPKα phosphorylation in the liver of rats with steatosis, which activating the Sirt1/AMPK signaling pathway (11). However, the potential effect of Oxymatrine in HFHF-induced obesity and the mechanism underlying Oxymatrine-mediated relief of metabolic disorders (such as obesity) remain unclear.

Here, we conducted a systematical investigation of Oxymatrine in HFHF-induced obesity. We applied an HFHF-diet rat model to investigate the therapeutic potential of Oxymatrine. In addition, we conducted ChIP analysis and luciferase-reported assays to examine the role of Oxymatrine in the epigenetic regulation of HFHF-diet-induced obesity.

## Materials and methods

### Animals study

40 Five- to six-week-old Wistar rats (176-185 g) were purchased from the Beijing Weitong Lihua Experimental Animal Co., Ltd. (Beijing, China). The animal studies were approved by the ethics committee of Hebei General Hospital and all animals received humane care in compliance with institutional animal care guidelines. After 7 days of acclimatization, Wistar male rats (6 weeks old) were randomly separated into three groups, the standard diet group (n=10) and the high-fructose and high-fat group (n=10), and the oxymatrine-treated high-fructose and high-fat group (n=10). Oxymatrine (Zhengdatianqing Pharmaceutic Company, Jiangsu, China) was dissolved in 0.5% carboxymethylcellulose sodium solution and was administered to the rat intragastrically.

### Cell culture

The 3T3-L1 adipocytes were purchased from the cell bank of the Chinese Academy of Sciences. The cells were cultured in Dulbecco’s Modified Eagle Medium (DMEM) (ThermoFisher Scientific) with 1% penicillin-streptomycin (10000 µ/ml), 10% fetal bovine serum (FBS), 10mmol/l L-glutamine, 1mmol/l sodium pyruvate. The cells were cultured in a humidified incubator (Thermo Scientific) containing 5% CO_2_ at 37°C. Routine detection of mycoplasma was negative.

### Chromatin Immunoprecipitation (ChIP)

Chromatin Immunoprecipitation experiments were conducted as previously reported (12). The subcutaneous adipocytes from 4 healthy or obese mice were pooled for ChIP-seq analysis. Briefly, the adipose tissue was dissected into small pieces and fixed in phosphate buffered saline (PBS) containing 1% formaldehyde for 15 minutes. The fixation was then quenched by adding 1/20 volume of 2.5 M glycine and washed twice in PBS containing 1% Triton X-100. The adipose tissue was ground in liquid nitrogen and washed three times in PBS containing 1% Triton X-100. The adipose tissue was sonicated in a Bioruptor Pico device for 25 minutes (30/30s on/off) in ChIP buffer. The ChIP experiments were then conducted according to the manufacturer’s instructions for the CHIP-IT high-sensitivity kit (Active Motif).

### ChIP-seq analysis

Through the Bowtie2 with default parameters, ChIP-Seq reads were mapped to the mouse reference genome (rn6). H3K27Ac peak calling was conducted by the Model-based Analysis of ChIP-Seq (MACS) program (version 1.4.2) with default settings. With a cut-off value of *p* < 10^-8^, peak calling was carried out for each sample, while the matched input genomic DNA was set as background control. The BedGraph file representing the map read count of a single sample was also generated using HOMER and uploaded to the university of california santa cruz (UCSC) Genome Browser for display. HOMER was used to generate a scatter plot. Use the annotate Peaks command with a size of 2000 and the log option to compare the peak SE differences between processed samples and untreated samples.

### High-throughput natural compounds screen

The natural compounds library was purchased from the MCE company (cat. HY-L021). A total of 30 plates (96-well) containing 2343 compounds were used in the current study. Dimethyl sulfoxide (DMSO) was used for negative control. Briefly, the 3T3-L1 cells were transfected with the pGL3-Bcl2 plasmid for 24 hours. Transfected cells were then seeded in the 384 culture plates and used for the screen. The luciferase activity was used for readout. Two replicates for each compound were used.

### Luciferase reporter analysis

A total of 1e4 3T3-L1 cells were seeded in a 24-well culture plate and transfected with the pGL3-Bcl2 and pRL-SV40 for 24 hours. 10mM oxymatrine was added post-transfection and cultured for additional 48 hours. The firefly luciferase activity and renilla luciferase activity were examined. The enhancer regions and corresponding promoter regions of Bcl2, Foxp1 and Ctnnb1 were cloned into the pGL3 luciferase vector (Promega) by Shanghai Dianxi Biotech. The firefly luciferase vector (200 ng) and Renilla luciferase expression vector (pRL TK) (5 ng) were transfected into mice adipocytes. According to the manufacturer’s instructions, cells were transiently co-transfected with luciferase report plasmid (Promega) and Lipofectamine 2000. After 48 hours of co-transfection with the designated plasmid, the double luciferase reporter assay system (Promega) was used. Renal luciferase, as an internal control, normalizes the transfection efficiency.

### Statistical analysis

Statistical analysis was conducted by SPSS v.22.0 or Prism GraphPad 8.0 software. Independent sample t-tests were carried out to calculate the *p*-values between the two groups. Analysis among multi-groups was performed using ANOVA. The *p*-value <0.05 served as a significant value. The experiments were repeated 3 times independently.

## Results

### High-throughput natural compounds screen identified oxymatrine inhibited the transcriptional activities of obesity-related genes

**Figure 1:**
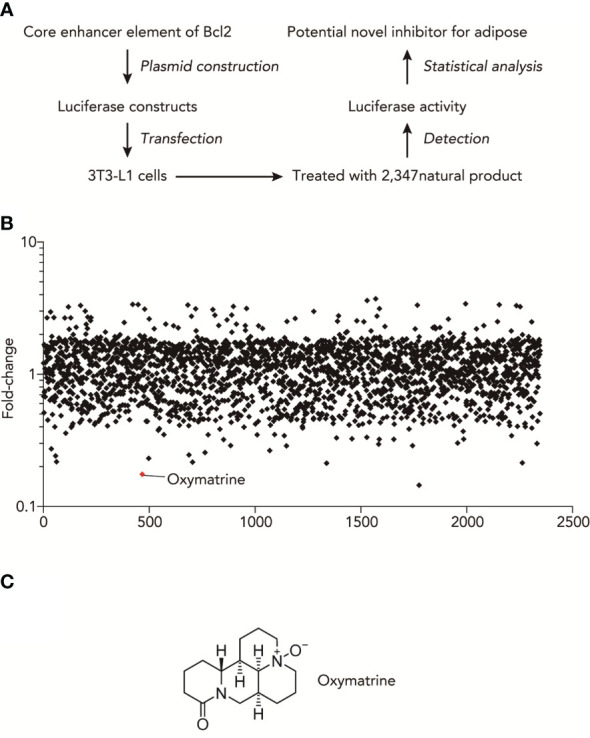
High-throughput natural compounds screen identified Oxymatrine inhibited the transcriptional activities of obesity-related genes. **(A)** Experimental design for Luciferase-based screen of natural compounds that could regulate the promoter activities of obesity-related genes. **(B)** Summary plot of promoter-activity inhibition screen, with Oxymatrine having very strong effects of inhibitory effects, as determined by folds changes of luciferase activity with or without the treatment of natural compounds. **(C)** Structures of Oxymatrine.

High-throughput luciferase activity screening was used to identify natural products that inhibited the transcriptional activities of obesity-related genes. We used a luciferase construct that contains an enhancer element of Bcl2, which showed the most difference between fructose and normal adipocytes. **Figure 1A** shows the experimental design for the luciferase-based screen of natural compounds that regulate the promoter activities of obesity-related genes. Based on the core enhancer element of Bcl2, luciferase was constructed and transfected into 3T3-L1 cells. Then, after treating with 2347 nature products, we detected the luciferase activity and found the potential novel inhibitor for adipose (**Table S1**). As shown in **Figure 1B**, the summary plot of the promoter activity inhibition screen was shown, which was determined by folds changes of luciferase activity with or without the treatment of natural compounds. Oxymatrine was identified because it showed a potent inhibitory effect (**Figure 1C**).

### Oxymatrine suppressed the HFHF-induced metabolic disorder

**Figure 2:**
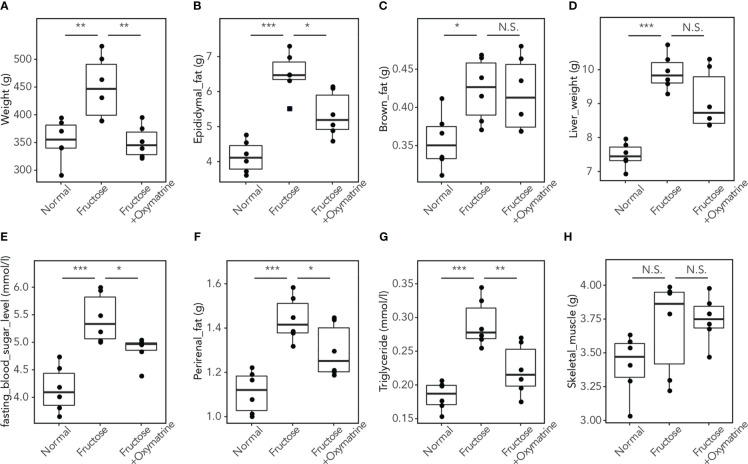
Oxymatrine inhibits the HFHF-induced high levels of fat, triglyceride, blood sugar, weight, and liver weight. The levels of weight **(A)**, epididymal fat **(B)**, brown fat **(C)**, liver weight **(D)**, blood sugar **(E)**, perirenal fat **(F)**, triglyceride **(G)**, and skeletal muscle **(H)** were detected in rat with a normal diet, HFHF-diet, and HFHF-diet with HFHF treatment. Results are expressed as the Mean ± SEM. *p < 0.05, **p < 0.01, ***p < 0.001, *N.S.* No significant compared with the control group.

To clarify whether Oxymatrine regulates the fructose-induced metabolic disorder *in vivo*, a high-fructose and high-fat (HFHF) diet rat model was used and treated with or without Oxymatrine. The levels of weight, epididymal fat, brown fat, liver weight, blood sugar, perirenal fat, triglycerides, and skeletal muscle were detected. As shown in **Figure 2**, compared to the HFHF diet, Oxymatrine decreased the HFHF-induced high levels of weight (449.03 ± 56.60 vs.350.83 ± 29.05g), epididymal fat (6.50 ± 0.61 vs. 5.34 ± 0.64 g), perirenal fat (1.43 ± 0.10 vs. 1.29 ± 0.12 g), blood sugar (5.43 ± 0.44 vs. 4.86 ± 0.25 mmol/l), and triglyceride (0.29 ± 0.03 vs. 0.22 ± 0.04 mmol/l), suggesting that Oxymatrine relieved the HFHF-induced metabolic disorder, especially the obesity symptoms *in vivo*.

### Oxymatrine altered the enhancer landscape of HFHF-diet

**Figure 3:**
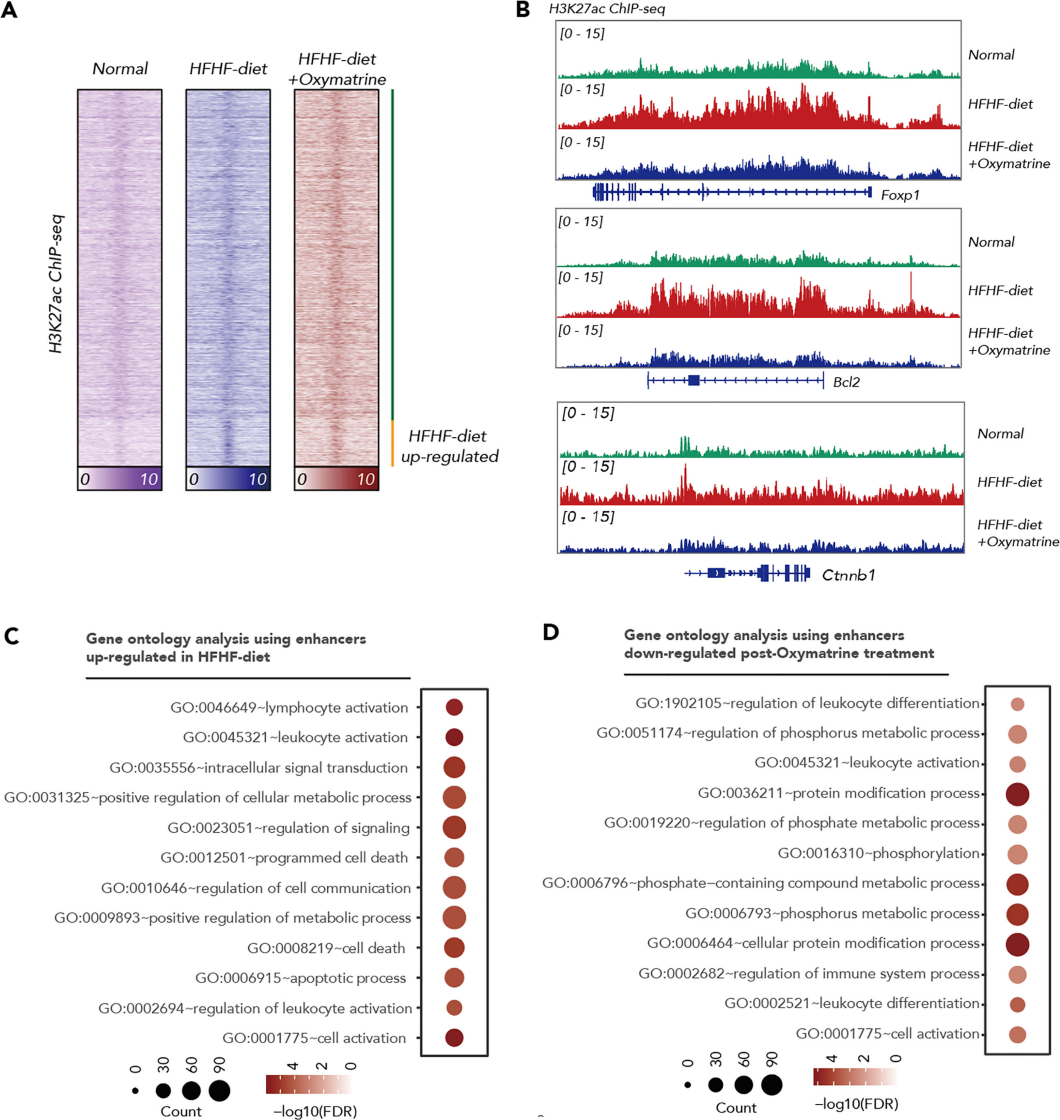
Oxymatrine alter the enhancer landscape of HFHF-diet. **(A)** Heatmap showing the H3K27ac signals in the subcutaneous adipose tissue of healthy rats, HFHF rats, and Oxymatrine-treated HFHF rats. The signals were shown by color. **(B)** ChIP-seq tracks of H3K27ac signals in subcutaneous adipose tissue of healthy rats, HFHF rats, and Oxymatrine-treated HFHF rats. Relative enrichment of H3K27ac signals in the regulatory regions for *Foxp1, Bcl2, and Ctnnb1* were shown. **(C)** Gene ontology analysis on enhancers altered post-HFHF diet. The size of each circle represents the number of enriched genes. The FDR value was shown by color. **(D)** Gene ontology analysis on enhancers altered post-Oxymatrine treatment. The size of each circle represents the number of enriched genes. The FDR value was shown by color.

Emerging evidence demonstrated that epigenetic alteration and transcriptional deregulation drives the progression of many diseases, including metabolic disorder. To address the potential regulatory function of Oxymatrine in HFHF-related metabolic disorders, we analyzed the enhancer profile of subcutaneous adipose tissue post-HFHF diet and Oxymatrine treatment. We conducted ChIP-seq analyses in subcutaneous adipose tissue isolated from healthy rats, HFHF-diet rats, and Oxymatrine-treated HFHF-diet rats, using antibodies against H3K27ac, a histone modification in active enhancer regions. A total of 33854 enhancers were identified in healthy rats, HFHF-diet rats, and Oxymatrine-treated HFHF-diet rats (**Figure 3A**). Most enhancers were shared among these three groups (**Figure S2**).

The acetylation of lysine 27 on histone H3 protein subunit (H3K27ac) signals was significantly increased in enhancers related to positive regulation of the metabolic process, lymphocyte activation, leukocyte activation, regulation of signaling, programmed cell death, and apoptotic process upon HFHF-diet (**Figures 3B, C**, **S2**). The H3K27ac signals were significantly decreased in enhancers related to the regulation of leukocyte differentiation, regulation of immune system process, regulation of phosphate metabolic process, and cellular protein modification process under Oxymatrine treatment (**Figures 3B, D**, **S2**). Most HFHF-diet-induced enhancers, such as *Bcl2*, Forkhead Box P1 (Foxp1), Catenin Beta 1 (Ctnnb1), Runt-related transcription factor 1 (Runx1), Kirsten rat sarcoma viral oncogene homolog (Kras), and CAMP responsive element binding protein 1 (*Creb1)* were suppressed upon Oxymatrine treatment (**Figures 3B**, **S3**).

### Oxymatrine altered the enhancer activity and expression of HFHF-related genes

**Figure 4:**
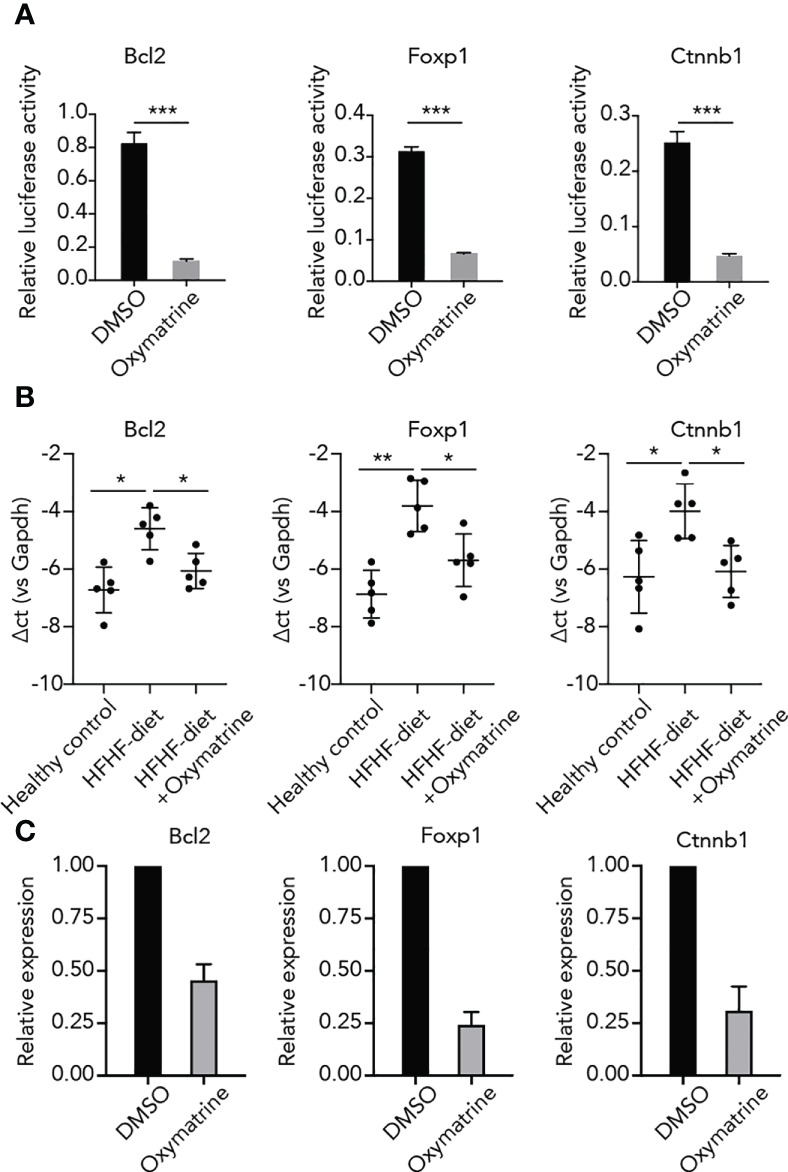
Oxymatrine suppressed the enhancer activity and expression of HFHF-related genes. **(A)** Oxymatrine suppressed the enhancer activity of Foxp1, Bcl2, and Ctnnb1. The Foxp1, Bcl2, and Ctnnb1 constructs were transfected into 3T3L1 cells with or without Oxymatrine treatment. **(B)** Oxymatrine suppressed the expression of Foxp1, Bcl2, and Ctnnb1 in subcutaneous adipose tissue. The expression of Foxp1, Bcl2, and Ctnnb1 was examined in subcutaneous adipose tissue of healthy rats, HFHF rats, and Oxymatrine-treated HFHF rats. **(C)** Oxymatrine suppressed the expression of Foxp1, Bcl2, and Ctnnb1 in 3T3-L1 cells. The expression of *Foxp1, Bcl2, and Ctnnb1* was examined in 3T3-L1 cells with or without Oxymatrine treatment. ^*^p < 0.05, ^**^p < 0.01, ^***^p < 0.001.

To further explore the regulatory function of Oxymatrine on HFHF-related enhancers, we examined the impact of Oxymatrine on the enhancer activity of HFHF-related genes. Three top differential enhancers between HFHF-diet and Oxymatrine-treated HFHF rats, including enhancers of Bcl2, Foxp1, and Ctnnb1, were selected for analysis (**Figures 1B**, **S2**). The enhancer activity of these three enhancers was all significantly suppressed post-Oxymatrine treatment (**Figure 4A**). In addition, we also examined the expression of these three genes in subcutaneous adipose tissue isolated from healthy rats, HFHF-diet rats, and Oxymatrine-treated HFHF-diet rats. The expression of these three genes was significantly up-regulated in HFHF-diet rats but was significantly down-regulated post-Oxymatrine treatment (**Figure 4B**). We further examined the expression of these three genes in 3T3-L1 cells, murine preadipocytes cell lines, with or without Oxymatrine treatment. Similar to that observed in the rat animal model, the expression of Bcl2, Foxp1, and Ctnnb1 was also significantly suppressed post-Oxymatrine treatment (**Figure 4C**).

### Oxymatrine suppressed the binding of Smad3 on HFHF-related genes

**Figure 5:**
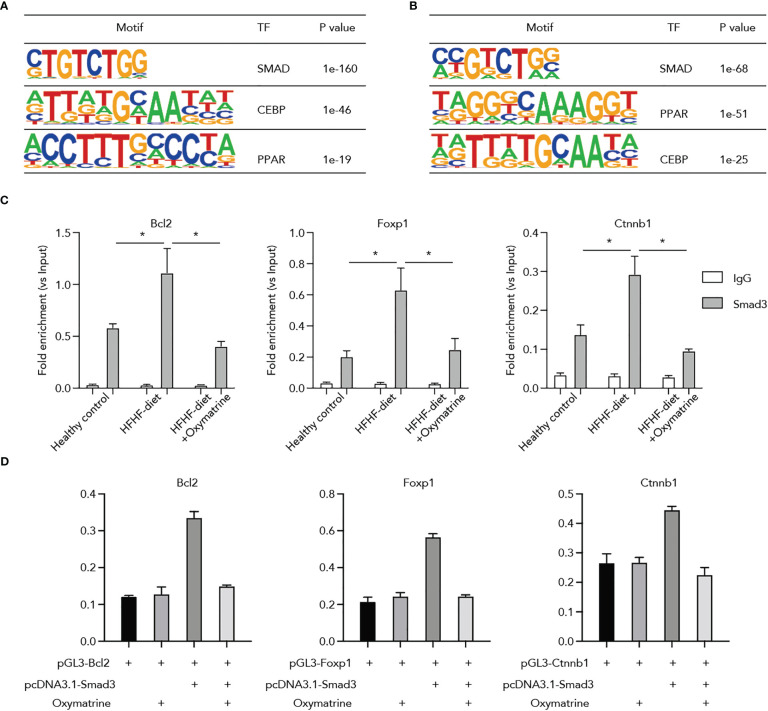
Oxymatrine suppressed Smad3 binding on the regulatory regions of Foxp1, Bcl2, and Ctnnb1. **(A)** Motif enrichment analysis of enhancers that altered post-HFHF diet. Enhancers were defined by the ChIP-seq signals of H3K27ac. Enhancers with a two-fold alteration between healthy and HFHF diets were used for motif enrichment analysis. **(B)** Motif enrichment analysis of enhancers that altered post-Oxymatrine treatment. Enhancers with a two-fold alteration between HFHF and Oxymatrine-treated HFHF rats were used for motif enrichment analysis. **(C)** Oxymatrine suppressed the binding of Smad3 on the enhancer regions of Foxp1, Bcl2, and Ctnnb1. ChIP-qPCR analysis was conducted in the subcutaneous adipose tissue of healthy rats, HFHF rats, and Oxymatrine-treated HFHF rats. **(D)** Smad3 enhanced the luciferase activity of *Foxp1, Bcl2, and Ctnnb1*, but the elevated enhancer activity could be suppressed by Oxymatrine. The *Foxp1, Bcl2, and Ctnnb1* constructs were co-transfected with the rat Smad3-expression plasmid and treated with or without Oxymatrine. * p <0.05.

Next, to ascertain potential targets of Oxymatrine in transcriptional regulation, we conducted a motif enrichment analysis using enhancers altered post-HFHF diet and under Oxymatrine treatment. SMADs motifs were significantly enriched in both enhancers altered post-HFHF diet and enhancers altered under Oxymatrine treatment (**Figure 5A**), suggesting a potential regulatory function of Oxymatrine SMAD transcription factor families, including Smad2, Smad3, Smad4, and Smad7. We examined the binding of these three transcription factors on the enhancer regions of Bcl2, Foxp1, and Ctnnb1, and only Smad3 a significant enrichment (**Figure S4**). To further explore the potential impact of Oxymatrine on Smad3, we analyzed the binding of Smad3 on the enhancer regions of Bcl2, Foxp1, and Ctnnb1 in subcutaneous adipose tissue isolated from healthy rats, HFHF-diet rats, and Oxymatrine-treated HFHF-diet rats (**Figure 5B**). The binding of Smad3 was significantly increased in the HFHF diet but was further suppressed under Oxymatrine treatment (**Figure 5C**).

Additionally, we analyzed the interplay between Oxymatrine and Smad3 on the enhancer activity of Bcl2, Foxp1, and Ctnnb1. We co-transfected the Bcl2, Foxp1, and Ctnnb1 luciferase constructs with the expression plasmids of Smad3 and treated the cells with or without Oxymatrine. The enhancer activities of Bcl2, Foxp1, and Ctnnb1 were significantly increased by the Smad3, but were suppressed by the Oxymatrine (**Figure 5D**), supporting the notion that Oxymatrine reprogramed the enhancer landscape of subcutaneous adipose tissues via suppressing the transcriptional activities of Smad3.

## Discussion

It is well proven that a high-fructose and high-fat diet (HFHF diet) can induce obesity, fatty liver disease and type 2 diabetes. Here, we identified that Oxymatrine, a natural product, showed potential in treating HFHF diet-induced obesity and metabolic disorder. We showed Oxymatrine suppressed the obesity-related features induced by HFHF diet, such as decreased body weight, perirenal fat, and liver triglyceride. We demonstrated HFHF-diet induced an alteration of enhancer landscape, such as increased the H3K27ac signaling of genes related to immune regulation, metabolic regulation, and cell apoptosis regulation. However, some of these enhancers could be suppressed by Oxymatrine. We further reported that the Oxymatrine suppressed the activity of enhancer activated by HFHF-diet via altering the binding of Smad3 on chromatin. Together, our study highlighted the regulatory function of Oxymatrine towards HFHF-diet-induced obesity and a potential application of oxymatrine in the treatment of obesity.

Obesity has led to a large economic burden and decreased quality of life (13). The long-term and excessive intake of dietary factors, such as high fructose and high fat, are highly related to the occurrence of obesity, fatty liver disease, and other metabolic disorders (14, 15). However, the role of HFHF diet on epigenetic alternations remains unclear. We here reported that the H3K27ac signaling on some enhancers related to immune response, apoptosis, and metabolic was upregulated. Drivers of obesity include the dysregulated immune response, suppressed apoptosis in adipose, and dysregulated lipids metabolisms. Thus, the altered enhancer landscape provided an additional layer of evidence that the HFHF diet also engaged in epigenetic changes of adipose.

Oxymatrine has been reported to exert multi-functions, including anti-oxidative stress, anti-inflammatory effects, anti/pro-apoptotic, anti-fibrotic, and metabolism regulatory effects (16). Oxymatrine also showed potential therapeutic effects in metabolic disorder-related diseases, such as non-alcoholic fatty liver disease (NAFLD) (16, 17). We previously showed that oxymatrine alleviated hepatic lipid metabolism via regulating microRNA-182 (miR-182) in the NAFLD model (17). Zhao et al. suggested that Oxymatrine may be the main active compound contributing to the lipid-lowering activity provided in NAFLD (18). Xu et al. demonstrated that Oxymatrine also can ameliorate hepatic steatosis (11). Oxymatrine is also reported to be a counterplay in transcription regulation, such as preventing the nuclear translocation of NF-κB (19).

Smad3 (SMAD Family Member 3) is a transcription factor involved in many diseases, including obesity and metabolic disorder. Lines of evidence demonstrated that Smad3 knockout mice protect mice from high-fat diet-induced obesity, diabetes, and insulin resistance (20–22). In addition, a previous study reported that inhibition of SMAD3 ameliorates obesity in rodents (23). We found that Smad3 binding was increased in HFHF-diet compared to healthy control, indicating that Smad3 might also be a driver in HFHF-diet-induced obesity. Moreover, we found that Oxymatrine suppressed the binding of Smad3 on chromatin and suppressed the activity of enhancers related to HFHF-diet in adipose tissue. Our study highlighted that the Oxymatrine showed a potential inhibitory function towards Smad3. Considering that Smad3 is also engaged in many other diseases, Oxymatrine might also be applied to other Smad3-related diseases.

Bcl2 encodes an integral outer mitochondrial membrane protein that blocks apoptotic death (24). The upregulation of Bcl-2 expression played an important role in apoptosis with fatty acid disease (25, 26). It was reported that Oxymatrine combined with Compound Yinchen Granules reduced the Bax/Bcl-2 ratio to inhibit the apoptosis of liver cells in acute liver failure. Another significant enhancer found in this study was Foxp1. Foxp1 belongs to subfamily P of the forkhead box (FOX) transcription factor family, which plays critical roles in the regulation of tissue gene transcription (27). A study showed that exosomes transferring miR-107 targeted the regulation of Foxp1 in clusters of differentiation 4 positive T (CD4+T) cells for the development of fatty liver disease (28). A recent study revealed the biological roles of Foxp1 in brown/beige adipocyte differentiation and thermogenesis, and overexpression of Foxp1 impairs adaptive thermogenesis and promotes diet-induced obesity in adipocytes (29). Furthermore, Adiposity and chronic inflammation are brought on by overeating, and white adipose tissue grows (WAT). The genetic variables governing fat mass and adiposity, however, are still largely unknown. It has been proven in a study that whole-exome sequencing in young obese participants and revealed rare gain-of-function mutations in CTNNB1/β-catenin linked with increased obesity risk. Attenuating the effects of a high-fat diet on obesity, sWAT mass expansion, and Pdgfr+ preadipocyte and mature adipocyte proliferation were achieved by specifically targeting-catenin in mature adipocytes (30). This study has significant clinical importance since this study highlighted the regulatory function of Oxymatrine towards HFHF-diet-induced obesity. Hence the application of oxymatrine could be a significant tool for the treatment of obesity. Nevertheless, there are some limitations in this study. It would be interesting to study in more detail the relevance of these results to human physiology.

## Conclusion

In this study, we systematically investigated the impact of oxymatrine on HFHF-induced obesity. This study highlighted oxymatrine could suppress high-fructose and high-fat diet-induced obesity by inhibiting Smad3 binding on obesity-related enhancers.

## Data availability statement

The datasets presented in this study can be found in online repositories. The names of the repository/repositories and accession number(s) can be found below: BioProject, PRJNA936626.

## Ethics statement

The animal study was reviewed and approved by the ethics committee of Hebei General Hospital.

## Author contributions

LR and SC conceived and designed the study. LR, HZ, WF analyzed the data. HZ, XY, and ZH performed the experiments. YZ performed visualization. LR and HZ wrote the paper. SC reviewed and edited the manuscript. All authors contributed to the article and approved the submitted version.
