# Supplementary material for: Oxymatrine relieves high-fructose/fat-induced obesity via reprogramming the activity of lipid metabolism-related enhancer
Source: Front Endocrinol (Lausanne). 2023 Aug 4;14:1145575. doi: 10.3389/fendo.2023.1145575 (PMC10437059; doi:10.3389/fendo.2023.1145575)
Supplement: Supplementary file 1 [file DataSheet_1.zip › supplementary materials/Supplementary Figures and Figure legends.docx]

**Supplementary Figures and Figure legends**


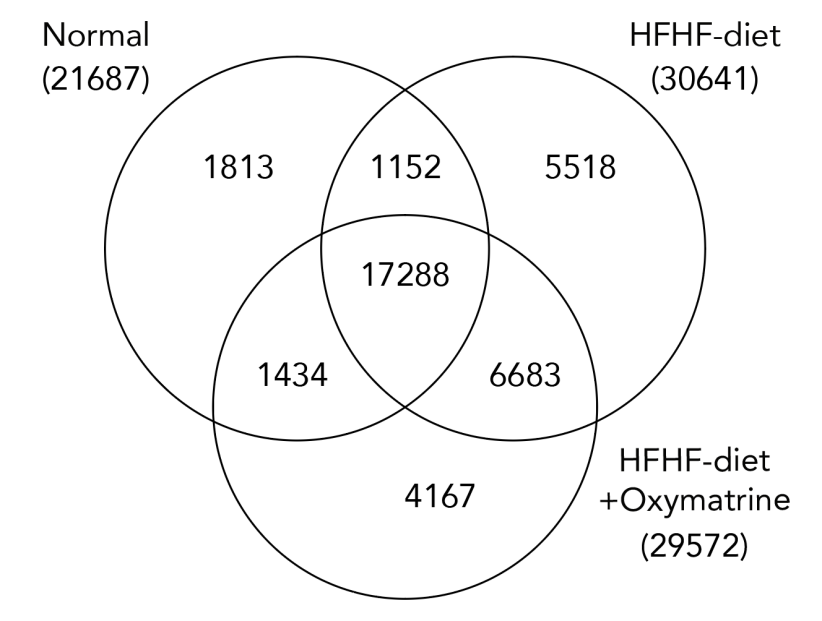


Figure S1. Overlaps among enhancers in healthy rats, HFHF rats, and Oxymatrine-treated HFHF rats.


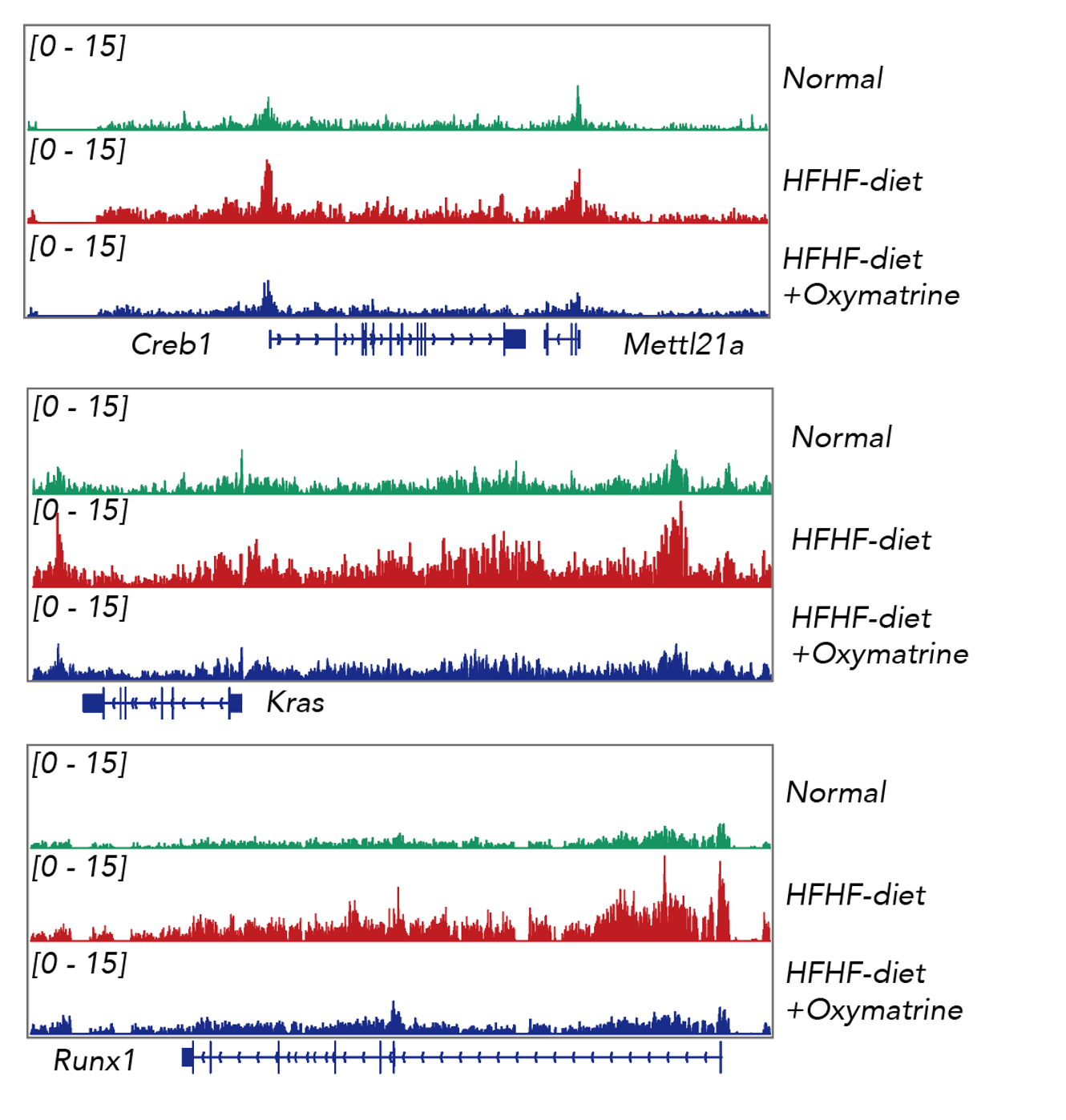


Figure S2. ChIP-seq tracks of H3K27ac signals in adipose tissue of healthy rats, HFHF rats, and Oxymatrine-treated HFHF rats. Relative enrichment of H3K27ac signals in the regulatory regions for *Creb1, Kras,* and *Runx1* were shown.


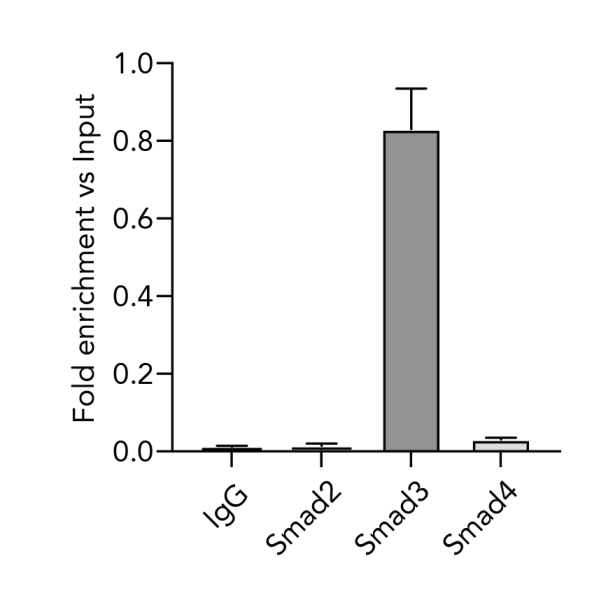


Figure S3. ChIP-qPCR analysis of Smad2/3/4/7 on the enhancer regions of Bcl2.

Table S1. H3K27ac ChIP-seq analysis of normal adipose tissue.

Table S2. H3K27ac ChIP-seq analysis of adipose tissue of HFHF rats.

Table S3. H3K27ac ChIP-seq analysis of normal adipose tissue of Oxymatrine-treated HFHF rats.
