# Supplementary figures and images for: Oxymatrine relieves high-fructose/fat-induced obesity via reprogramming the activity of lipid metabolism-related enhancer
Source: Front Endocrinol (Lausanne). 2023 Aug 4;14:1145575. doi: 10.3389/fendo.2023.1145575 (PMC10437059; doi:10.3389/fendo.2023.1145575)

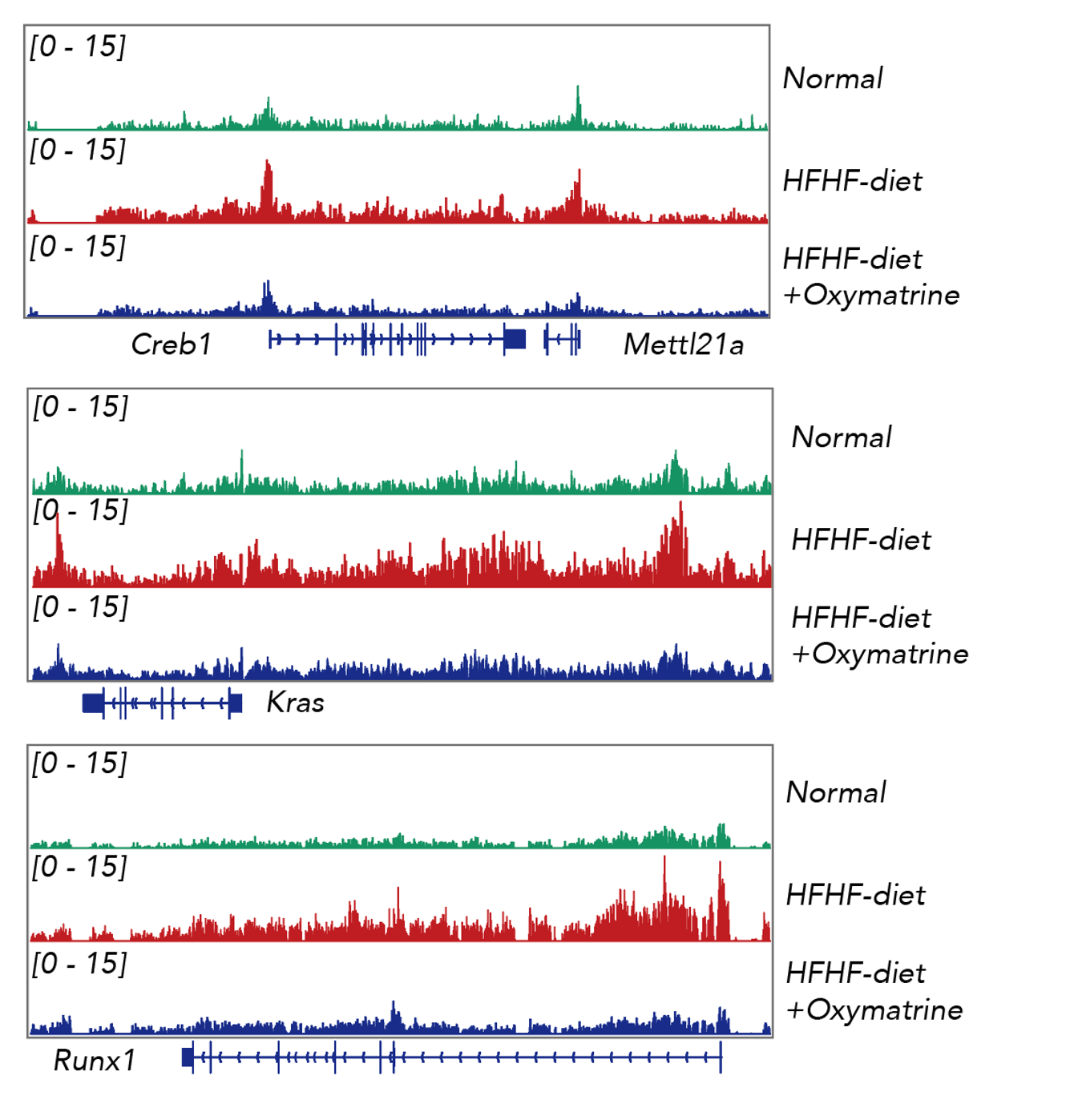

Supplement: Supplementary file 1 [file DataSheet_1.zip › supplementary materials/Revised Figure S2.tif]
